# Stereotactic radiosurgery for arteriovenous malformations presenting as secondary trigeminal neuralgia: a case series

**DOI:** 10.1007/s10143-025-03400-9

**Published:** 2025-02-17

**Authors:** Ali Haluk Düzkalir, Mehmet Orbay Askeroglu, Selcuk Peker

**Affiliations:** 1 Department of Neurosurgery, Koç University Hospital, Istanbul, Turkey; 2 Department of Neurosurgery, Gamma Knife Center, Koç University Hospital, Istanbul, Turkey; 3 Department of Neurosurgery, Koç University School of Medicine, Istanbul, Turkey; 4 Koç University Hospital, Davutpasa St., No: 4, Topkapi, Istanbul, 34010 Turkey

**Keywords:** Stereotactic radiosurgery, Trigeminal neuralgia, Arteriovenous malformation

## Abstract

Stereotactic radiosurgery (SRS) is a potential treatment for trigeminal neuralgia (TN) secondary to arteriovenous malformations (AVMs), though its efficacy and safety remain unclear due to the rarity of this condition. We analyzed 1211 brain AVM cases treated with Gamma Knife radiosurgery (GKRS) between 2005 and 2023 at our institution. Four patients (0.33%) were presented with TN secondary to AVM. Three patients received single-fraction GKRS while one underwent hypofractionated treatment. Treatment outcomes were assessed using magnetic resonance imaging, digital subtraction angiography, and the Barrow Neurological Institute pain intensity scale. The mean marginal dose and AVM volume were 21.5 Gy and 0.58 cc, respectively. The mean follow-up period was 85.75 months. Complete AVM obliteration was achieved in all patients, with all experiencing complete pain relief within a mean time of 18 months, enabling gradual discontinuation of medications. No radiation-related adverse effects were observed. Our literature review identified only 15 previously reported cases where SRS was used as primary treatment for TN secondary to AVM, with most cases showing favorable outcomes in pain relief and AVM obliteration. This study is the first case series to demonstrate the sole use and efficacy of GKRS in managing TN secondary to AVM, moving beyond individual case reports. SRS appears to be a safe and effective primary treatment option for TN secondary to AVM, particularly when conventional surgical approaches are contraindicated or pose excessive risks. The sustained pain relief and absence of complications in our series, combined with previous case reports, support its use in managing this rare condition.

## Introduction

Trigeminal neuralgia (TN) is characterized by “recurrent unilateral brief electric shock-like pains, abrupt in onset and termination, limited to the distribution of one or more divisions of the trigeminal nerve and triggered by innocuous stimuli” [21]. Although the pathogenesis of TN is not fully understood, the most widely accepted theory presumes that the trigeminal sensory fibers within the proximal nerve root become demyelinated [23].

Classic TN is related to vascular compression of the trigeminal nerve root frequently identified on magnetic resonance imaging (MRI) as morphological changes. Secondary TN is caused by an underlying disease, with the most common causes including multiple sclerosis, tumors, skull-base bone deformity, connective tissue diseases, arteriovenous malformations (AVMs), dural arteriovenous fistulas, and genetic causes of neuropathy [2].

Posterior fossa AVMs are uncommon, accounting for 7–15% of all intracranial AVMs. It is extremely rare for a posterior fossa AVM to present with TN, and very few cases have been published in the English literature [1, 3, 5, 11, 13, 20].

This case series provides insights into the potential role of stereotactic radiosurgery (SRS) for managing TN caused by AVMs, a rare and complex condition, and aims to contribute to the evolving understanding of optimal treatment strategies for these challenging cases.

## Materials and methods

**Fig. 1:**
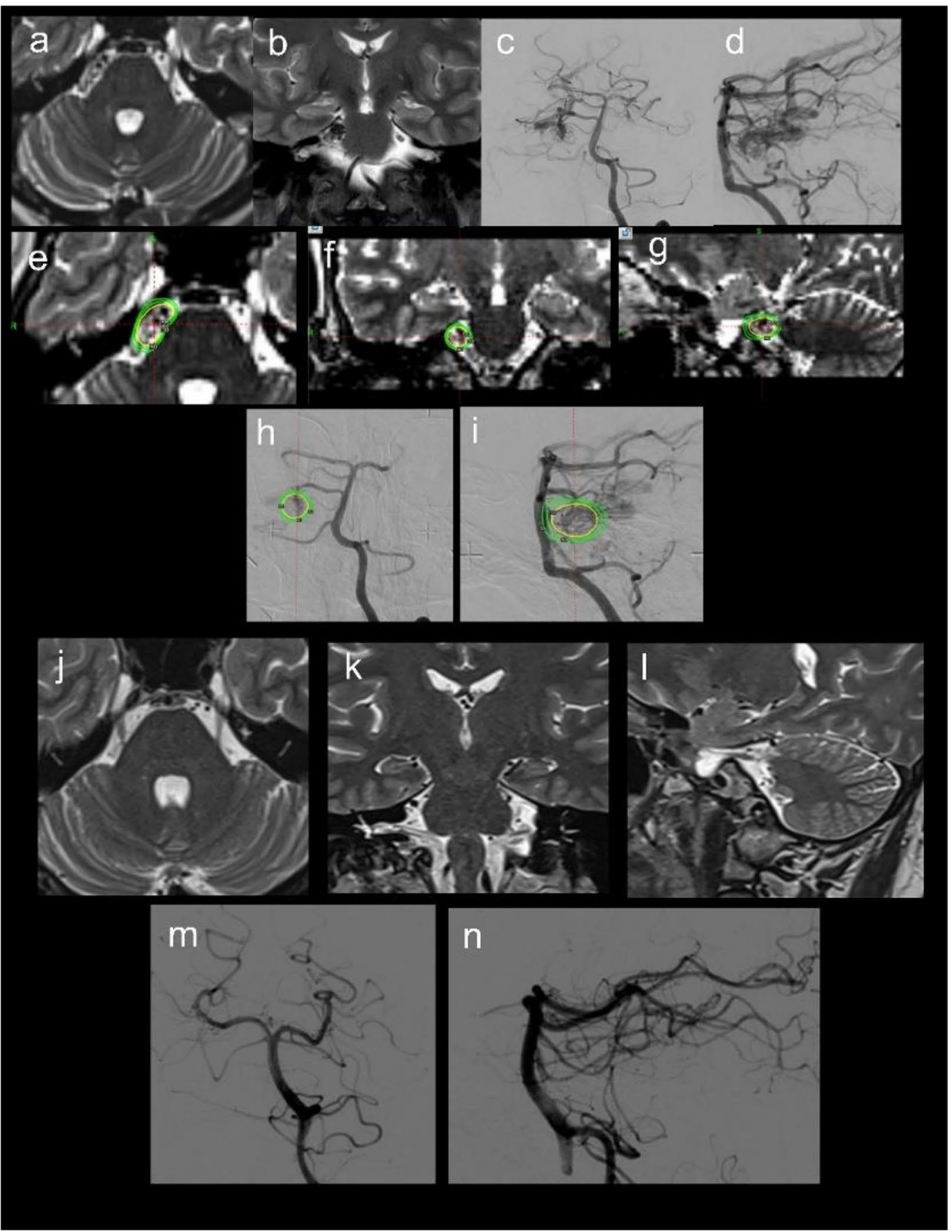
Imaging studies of a patient presenting with TN on the day of GKRS. Axial T2-weighted MR (**a**) and coronal T2-weighted MR (**b**) images demonstrate a right ponto-cerebellar AVM. Anterior-posterior (**c**) and lateral (**d**) cerebral DSA images show the AVM supplied by the right superior cerebellar artery. (**e**-**i**) GKRS planning images. The AVM was treated with a marginal dose of 18 Gy at the 55% isodose line. (**j**-**n**) Follow-up MR and DSA images illustrating complete obliteration of the AVM at the time of last follow-up

Of 1211 brain AVM cases treated with GKRS in our clinic between 2005 and 2023, only 4 patients (0.33%) had TN secondary to AVM. MRI and cerebral digital subtraction angiography (DSA) revealed that each patient had an AVM that was in direct contact with the trigeminal nerve, causing neuralgia. Leksell Gamma Knife Perfexion and Icon (Elekta, Sweden) were used for radiosurgical treatment. Three patients, who received single-session Gamma Knife radiosurgery (GKRS), underwent stereotactic DSA with a Leksell Coordinate Frame G (Elekta, Sweden) prior to treatment. Axial T1, post gadolinium enhanced axial T1, axial T2, axial MR angiography and computed tomography (CT) images were collected with a slice thickness of 1–2 mm. BrainLab™ Elements (BrainLab, Inc., Munich, Germany) software was utilized to correct MRI distortions using CT images. The distortion-corrected MR images were then fused with cerebral DSA images to delineate the target within the Leksell GammaPlan treatment planning system (Fig. 1). In one patient who underwent hypofractionated treatment, the same imaging procedures were performed except for stereotactic DSA. All treatments were targeted to the AVM nidus and no SRS was performed for the trigeminal nerve. For follow-up, the first MRI was performed 6 months after GKRS and annually thereafter. At the end of the third year of treatment, cerebral DSA was performed in all cases to evaluate the presence of a residual AVM that could not be seen on MRI. The Barrow Neurological Institute (BNI) pain intensity scale was used to compare pain before and after GKRS in a retrospective review of medical records [16].

## Results

All patients were male. The mean age was 50.5 (range, 31–69 years) at the time of treatment. Typical TN was the sole and primary presenting symptom for all patients. All had received prior drug treatment without success when the AVM was identified during the evaluation of the TN. None of the patients had experienced any bleeding episodes pre- or post-GKRS.

In two cases, the AVM was located in the cerebellopontine angle (CPA), compressing the root entry zone (REZ) of the trigeminal nerve. In one case, it was located in the superior cerebellar artery distribution, surrounding the entire cisternal segment of the trigeminal nerve and contacting the REZ. In another case, it was in the lateral cerebellomedullary cistern, adjacent to the mid-segment of the trigeminal nerve.

The mean follow-up was 85.75 months (range, 48–107). Three patients underwent single fraction, and one patient underwent hypofractionated treatment. Two of the patients treated in single session received a marginal dose of 18 Gy and one received a marginal dose of 20 Gy. For one patient (case No. 3), the AVM nidus was in close proximity to the brainstem, presenting a significant challenge in radiosurgical planning. Initial treatment planning revealed that a single-fraction dose of 18–20 Gy would result in excessive radiation exposure to the brainstem, exceeding the 14 Gy safety threshold. To mitigate this risk, we employed a hypofractionated, thermoplastic mask-based GKRS approach, delivering the treatment over five consecutive days. A marginal dose of 6 Gy per fraction was applied, resulting in a cumulative dose of 30 Gy to the AVM nidus while keeping the brainstem dose within acceptable limits.

The mean marginal dose was 21.5 Gy (range, 18–30 Gy), and the maximum dose was 41.4 Gy (range, 32.7–60). The mean AVM volume was 0.58 cc (range, 0.21–1.52 cc).

Last follow-up MRI and DSA examinations demonstrated complete obliteration of the AVM in all patients (Fig. 1). Prior to the third year, MRI follow-ups revealed gradually shrinking AVMs, but obliteration did not appear until the third-year DSA.

All patients experienced complete relief of pain following treatment, leading to a gradual discontinuation of medications. The mean duration of complete pain relief following GKRS was 18 months (range, 12–24). None of the patients had early or delayed adverse effects due to radiation. Demographics, main clinical and treatment characteristics, obliteration outcomes, and BNI scores before and after treatment are shown in Table 1.

**Table 1:** Demographics, main clinical and treatment characteristics, obliteration outcomes, and BNI scores before and after treatment of our series

Patient No.	Age (years), sex	Pain location (side, division)	AVM location	TN duration before SRS (months)	Prior treatment	BNI before SRS	AVM volume (cc)	SRS modality	Isodose line (%)	Marginal dose (Gy)	No. of fractions	Dmax (Gy)	Follow-up (months)	Complete pain relief	Time to complete pain relief (months)	BNI at last follow-up	Medication at last follow-up	Obliteration	Complications
1	31/M	R, V3	R, CPA	18	CBZ 800 mg/day	IV	0,21	GKRS	55	18	1	32,7	87	Yes	12	I	No	Obliterated on 3rd year DSA after treatment	No
2	69/M	R, V2-3	R, CPA	22	CBZ 800 mg/day	IV	0,28	GKRS	50	20	1	40	101	Yes	18	I	No	Obliterated on 3rd year DSA after treatment	No
3	44/M	L, V2-3	L, CPA	6	CBZ 1200 mg/day	IV	1,52	GKRS	50	30 (5 × 6)	5	60	48	Yes	18	I	No	Obliterated on 3rd year DSA after treatment	No
4	54/M	R, V2	R, CPA	10	CBZ 800 mg/day, PGB 300 mg/day	IV	0,33	GKRS	55	18	1	32,7	107	Yes	24	I	No	Obliterated on 3rd year DSA after treatment	No

Notes: CBZ: Carbamazepine; PGB: Pregabalin

## Discussion

Due to the rarity and heterogeneity of this entity, the optimum treatment for TN induced by AVM is uncertain. If possible, microsurgery is a good option for completely removing the AVM and decompressing the trigeminal nerve. However, dilated vessels and arterialized veins may restrict the surgical corridor, compress the nerve, and rupture to cause massive bleeding, increasing the risk of intraoperative and postoperative complications in TN secondary to AVM.

The main goal of SRS is to achieve total nidus obliteration in order to prevent further brain hemorrhage and potential adverse effects in AVMs. Particularly for AVMs that are small and located in surgically risky areas, SRS has become the treatment of choice due to its high obliteration rate and low mortality rate [4]. However, for TN induced by AVM, embolization and/or microvascular decompression (MVD) is often preferred in the literature [6, 8, 9, 12, 18, 23].

TN secondary to AVM is an exceptionally rare condition, with only a limited number of cases documented in the literature. In 2016, Yuan et al. reported 40 cases of TN secondary to brain AVMs across 29 articles [23]. In 2017, Li et al. reported only 15 cases of TN secondary to cerebellar AVMs over the past 56 years, including their own case [11]. Although reports of TN secondary to AVM are exceedingly uncommon, publications focusing primarily on the use of SRS for its treatment are even more scarce. To the best of our knowledge, only 15 cases have been reported in which SRS was primarily used in the treatment for TN secondary to AVM (Table 2).

**Table 2:** Reported cases of TN secondary to AVM

Author(s)	Age (years), sex	Pain location (side, division)	Prior treatment	BNI before SRS	AVM volume (cc)	SRS modality	Isodose line (%)	Marginal dose (Gy)	No. of fractions	Dmin, Dmax (Gy)	Follow-up (months)	Complete pain relief	Time to complete pain relief (months)	BNI at last follow-up	Medication at last follow-up	Obliterated	Complications
**Sato et al.** [15]	49/F	R, V2	N/A	N/A	N/A	GKRS	N/A	N/A	N/A	N/A	24	Not relieved after GKRS. MVD was performed 1 year after GKRS.	N/A	I	No	No	No
**Anderson et al.** [3]	39/M	R, V2	CBZ 1500 mg/day, PST 325 mg/day, OXY 10 mg/day	N/A	0,89	GKRS	50	20,1	1	N/A	13	Yes	13	I	Stopped at the last follow-up	N/A	No
**Matsushige et al.** [12]	50/M	R, V2-3	No	N/A	N/A	GKRS	60	30	1	N/A	36	Yes	12	I	No	N/A	No
**Sumioka et al.** [17]	66/M	R, V3	MVD	N/A	N/A	GKRS	N/A	N/A	N/A	N/A	18	Complete pain relief achieved after MVD. GKRS was then applied for AVM.	Immediately relieved after MVD	I	N/A	Yes	No
**Mori et al.** [13]	69/M	L, N/A	Embolization, CBZ (dose is not specified)	N/A	5,4	GKRS	N/A	20	1	N/A	72	Yes	18	I	No	No	No
**Cho et al.** [5]	N/A	N/A	N/A	III	N/A	GKRS	50	32	1	N/A	36	Yes	N/A	I	N/A	Yes	No
**Isik et al.** [9]	47/M	L, V2-3	Embolization, CBZ (dose is not specified)	N/A	N/A	GKRS	50	15	1	N/A	18	Yes	18	I	N/A	No	No
**Ahmed et al.** [1]	56/M	L, V3	CBZ and LTG, doses are not specified	N/A	N/A	GKRS	N/A	20 to nidus, 85 to trigeminal nerve REZ	1	N/A	12	No (considerably reduced, but not completely relieved)	N/A	N/A	N/A	N/A	No
**Tjahjadi et al.** [18]	39/F	R, V2-3	N/A	N/A	N/A	GKRS	45 for nidus, 100 for trigeminal nerve REZ	20 to nidus, 80 to trigeminal nerve REZ	1	N/A	60	Not relieved after GKRS. MVD was performed 18 months after GKRS.	Immediately relieved after MVD	I	No	No	No
**Yang et al.** [20]	68/F	N/A, V2	Rhizotomy, CBZ (dose is not specified)	N/A	0,4	CK	N/A	18	1	N/A-25,7	100	Yes	3	I	No	Yes	N/A
	39/M	N/A, V2	No	N/A	0,89	GKRS	N/A	20	1	N/A-40	203	Yes	6	I	N/A	Yes	N/A
	36/F	N/A, V2	CBZ and GBP, doses are not specified	N/A	0,31	GKRS	N/A	14	1	N/A-28	26	Yes	6	I	No	Yes	N/A
	12/M	N/A, V1	No	N/A	1,18	CK	N/A	20	1	N/A-28,1	45	Yes	12	I	N/A	Yes	N/A
	47/F	N/A, V2-3	GBP, dose is not specified	N/A	0,31	CK	N/A	20	1	N/A-27,3	50	Yes	0,7	I	No	No	N/A
	46/M	N/A, V2	MVD, CBZ (dose is not specified)	N/A	0,26	CK	N/A	16	1	N/A-23,5	33	Yes	11	I	No	Yes	N/A

Notes: CBZ: Carbamazepine; PST: Paracetamol; OXY: Oxycodone; LTG: Lamotrigine; GBP: Gabapentin

In 2003, Sato et al. reported a case in which the patient initially underwent GKRS, which partially reduced the size of the AVM but did not alleviate the TN [17]. One year after GKRS, a subsequent MVD successfully decompressed the trigeminal nerve, leading to complete pain relief. At the last follow-up, 24 months after GKRS and 12 months after MVD, the patient remained pain-free without the need for medication. The authors hypothesize that the reflux from the AVM caused the veins to become “arterialized,” mimicking the pulsatile compression of an artery. GKRS treatment in this case targeted the nidus of the AVM, while the authors did not provide specific parameters for the treatment.

In 2006, Anderson et al. reported a case of AVM-induced TN that was successfully treated with GKRS, with a dose of 20.1 Gy prescribed to the 50% isodose line [3]. This was the first case report where radiosurgery was the sole treatment for an AVM presenting with TN. They reported a 6-month follow-up MRI that revealed a reduction in the volume of the flow void abnormality, and the patient was no longer having painful episodes and had stopped carbamazepine treatment at 13 months of follow-up.

Matsushige et al. reported a case of dural AVM, presenting as TN, that underwent solely GKRS treatment [13]. The dosimetry plan aimed to cover the 0.3 cc volume, administering a dose of 30 Gy to the 60% isodose line. Follow-up MRI showed a thrombosed lesion, and the patient experienced relief from TN and maintained medication control for one-year post-treatment. They reported that the patient has been free of the symptom, and there has been no evidence of recurrence during a follow-up period of 3 years.

In 2011 Sumioka et al. reported a case of TN secondary to an AVM embedded within the trigeminal nerve [19]. The patient opted for direct surgical treatment to achieve immediate pain relief. During surgery, authors observed that the nidus of the AVM was almost completely embedded in the proximal part of the sensory root of the trigeminal nerve, and the right superior cerebellar artery (SCA) was compressing the nerve at the REZ. After successfully transpositioning the SCA and achieving immediate pain relief, the patient underwent GKRS one month later to treat the AVM’s nidus, which was no longer visible on MRI eighteen months after SRS. The authors did not provide specific data about the SRS treatment parameters, such as the dose, isodose line, or target volume used for this patient. Although this case was reported as TN secondary to AVM, MVD was chosen as the primary treatment for TN, followed by GKRS for AVM treatment. However, the finding of the complete disappearance of the AVM on an MRI eighteen months later and the relief of pain in the patient can be considered an indicator of the efficacy of GKRS treatment.

In 2014, Mori et al. reported a case in which the patient underwent embolization of the AVM, which resulted in a reduction in the size of the AVM and some improvement in his symptoms. After one month, the patient underwent GKRS to further reduce the size of the residual AVM. The nidus, measuring 5.4 cc, was treated with 20 Gy, leading to complete resolution of TN 1.5 years after GKRS. Six years post-GKRS, the patient remained pain-free without medication, and MRI showed a remarkable reduction in the abnormal vessels surrounding the nidus. The authors suggested that embolization followed by GKRS may be a less invasive and effective treatment option for TN caused by AVM.

Cho et al. conducted a retrospective study analyzing 50 patients with secondary TN treated with GKRS between 2002 and 2011, encompassing various lesion types and locations [5]. Notably, only one case involved TN secondary to an AVM. In this case, 32 Gy was applied to the nidus at the 50% isodose line, the nidus was obliterated after three years, and the patient’s BNI score improved from III to I.

Işik et al. reported a case of TN secondary to an AVM that was treated with embolization 18 years earlier; however, the procedure failed to achieve complete obliteration [10]. Despite long-term medication use, which provided partial relief, the patient’s pain persisted. They treated the nidus with a dose of 15 Gy prescribed to the 50% isodose line. They reported that, although the AVM was not completely obliterated 1.5 years after treatment, the pain had completely resolved.

Ahmed et al. presented a case of TN caused by an AVM in the left cerebellopontine angle cistern [1]. Despite initial medical treatment, the patient’s pain persisted, highlighting the limited efficacy of standard pharmacological approaches in such cases. GKRS was employed, delivering a dual-targeted approach: a regular radiation dose of 20 Gy to the AVM nidus and a higher dose of 85 Gy to the trigeminal nerve REZ. Follow-up at one year revealed significant pain relief, with the patient reporting only occasional mild discomfort that did not interfere with daily activities. They reported that they have not yet performed follow-up radiologic imaging, so there is no data on the final extent of the AVM. Furthermore, the application of a separate GKRS to the REZ of the trigeminal nerve may have contributed to the patient’s actual benefit, although the efficacy of both targeted therapies remains uncertain.

Tjahjadi et al. reported two cases of TN secondary to AVM. They initially treated one of them with GKRS, administering 20 Gy to the nidus and 85 Gy to the REZ of the trigeminal nerve; however, despite a significant obliteration of the AVM, the patient did not benefit from the treatment and underwent MVD at the end of the 18th month [20]. The other case did not receive any treatment other than MVD. They reported that the patient who underwent GKRS was still free of pain and medication at 60 months follow-up.

Yang et al. recently conducted a comprehensive analysis of eight cases of TN associated with peripontine AVMs [22]. Of these, four patients were treated using CyberKnife (CK), two with GKRS, and two were managed conservatively. In their study, the six patients who underwent SRS targeting the AVM nidus received an average dose of 1800 cGy, a mean maximum dose of 2879.895 cGy, and an average target volume of 0.563 cc. This approach resulted in complete resolution of TN symptoms in all treated cases, with a mean time to symptom resolution of 193 days (range: 21–360 days). Moreover, AVM obliteration was achieved in all, but one patient treated with CK. The average follow-up period for these patients was reported to be 5.4 years, during which all individuals were able to discontinue TN medications, underscoring the effectiveness and lasting impact of SRS in treating TN associated with AVMs.

GKRS has become more popular as a less invasive option for treatment of brain AVMs, particularly for patients who are not ideal candidates for surgical excision due to the high risk of bleeding or other complications. However, there are instances where GKRS may not achieve sufficient obliteration of the AVM, necessitating alternative approaches such as embolization and MVD. In our series, primary GKRS was effective in achieving both pain relief and AVM obliteration in all cases, without requiring subsequent embolization or MVD. This outcome supports the use of GKRS as a primary treatment modality, even for patients with medically intractable and intolerable neuralgia. GKRS offers the advantage of being less invasive, with a favorable safety profile compared to MVD or embolization, particularly in high-risk cases. However, in patients who experience delayed pain relief or incomplete AVM obliteration, the timing and necessity of adjunctive procedures should be carefully evaluated on a case-by-case basis.

The determination of the optimum dose and fractionation scheme in GKRS for AVMs remains a critical factor in achieving effective nidus obliteration while minimizing adverse effects. Studies show that single-session SRS with marginal doses between 18 and 25 Gy results in high obliteration rates for AVMs [7, 14]. However, for larger AVMs or those located near critical structures, single-session treatments may carry a higher risk of radiation-induced complications due to the dose-volume effect. The α/β ratio of AVM target cells, typically ranging from 2 to 3, is higher than that of the surrounding tissues, making fractionation an effective and appropriate treatment approach [15]. Since exposure of the brainstem to more than 14 Gy can lead to adverse radiation effects and new neurological deficits, we delivered the treatment in five fractions of 6 Gy each in one of our cases where the AVM was located near the brainstem.

In our study, AVM obliteration and complete pain relief did not occur simultaneously in any of the cases. For all four patients, MRIs performed at the time of complete pain relief still showed the presence of the AVM nidus, indicating that AVM obliteration occurred later. This finding suggests that the disappearance of trigeminal pain is not directly dependent on the complete obliteration of the AVM. Instead, it may be attributed to indirect effects of radiosurgery, such as reduced vascular pulsations or changes in the hemodynamics of the trigeminal nerve region.

Most of the very limited number of case reports in the literature on the treatment of AVM secondary to TN with SRS lack valuable data such as the use of objective pain scoring, the volume of the AVM, DSA evaluation demonstrating total obliteration, and SRS parameters, which are important in evaluating post-treatment outcomes. However, with the exception of three cases [1, 17, 20] in the literature where SRS was applied as an initial treatment, it seems to have been successful in relieving TN symptoms in the other 16 cases, including ours. Additionally, the mean follow-up period for cases in the literature, including the present study, where SRS was utilized as the initial treatment option, was 57.31 months (range: 12–203 months). The sustained pain relief and lack of significant radiation-related side effects observed during this period further highlight the efficacy and safety of SRS in managing this patient population.

### Limitations

Despite the promising outcomes observed in this study, several limitations must be acknowledged. Firstly, the sample size is small, with only four cases, which restricts the generalizability of our findings. However, the main reason for this is that cases of TGN secondary to AVM are exceedingly rare. Additionally, the retrospective design of this study, depending solely on medical records, may introduce bias and limit the reliability of the data collected.

Another limitation is the absence of a control group or direct comparison with other treatment modalities, such as MVD or endovascular embolization, which makes it challenging to assess the relative efficacy of GKRS for TN secondary to AVM. Also, the variability in radiosurgical parameters, such as dose and fractionation schedule, further complicates determining the best SRS protocol for TN secondary to AVM. Although the study offers comprehensive dosimetric data, standardizing recommendations can be challenging due to patient heterogeneity in treatment approaches.

Finally, although the mean follow-up was 85.75 months, larger studies with longer follow-up are needed to confirm the durability of pain relief in this exceedingly rare and specific entity and to evaluate the potential late-onset side effects of GKRS.

## Conclusion

The application of SRS for managing TN secondary to AVM is exceptionally rare in the existing literature. To our knowledge, this is the first case series to demonstrate the use and efficacy of GKRS solely in the management of TN secondary to AVM, derived from a cohort of patients rather than a case report. The observed obliteration rates, sustained symptom relief, and absence of significant adverse effects reinforce the potential of SRS as a primary therapeutic modality, especially in cases with TN secondary to AVM where conventional surgical approaches are contraindicated or pose excessive risks. Nevertheless, further studies and larger series are needed to assess the long-term outcomes and potential complications associated with this treatment approach. Comparative studies between SRS and alternative therapeutic strategies could also provide critical insights for optimizing the management of TN secondary to AVM.

## Data Availability

No datasets were generated or analysed during the current study.
